# A Brief Review of Molecular Techniques to Assess Plant Diversity

**DOI:** 10.3390/ijms11052079

**Published:** 2010-05-10

**Authors:** Ibrahim A. Arif, Mohammad A. Bakir, Haseeb A. Khan, Ahmad H. Al Farhan, Ali A. Al Homaidan, Ali H. Bahkali, Mohammad Al Sadoon, Mohammad Shobrak

**Affiliations:** Molecular Fingerprinting and Biodiversity Unit, Prince Sultan Research Chair for Environment and Wildlife, College of Sciences, King Saud University, Riyadh, Saudi Arabia

**Keywords:** molecular diversity, DNA fingerprinting, plant species, conservation genetics

## Abstract

Massive loss of valuable plant species in the past centuries and its adverse impact on environmental and socioeconomic values has triggered the conservation of plant resources. Appropriate identification and characterization of plant materials is essential for the successful conservation of plant resources and to ensure their sustainable use. Molecular tools developed in the past few years provide easy, less laborious means for assigning known and unknown plant taxa. These techniques answer many new evolutionary and taxonomic questions, which were not previously possible with only phenotypic methods. Molecular techniques such as DNA barcoding, random amplified polymorphic DNA (RAPD), amplified fragment length polymorphism (AFLP), microsatellites and single nucleotide polymorphisms (SNP) have recently been used for plant diversity studies. Each technique has its own advantages and limitations. These techniques differ in their resolving power to detect genetic differences, type of data they generate and their applicability to particular taxonomic levels. This review presents a basic description of different molecular techniques that can be utilized for DNA fingerprinting and molecular diversity analysis of plant species.

## 1. Introduction

The conservation and sustainable use of plant genetic resources require accurate identification of their accession. The emergence of DNA-based markers has changed the practice of species identification techniques [1]. The dramatic advances in molecular genetics over the last few years have provided workers involved in the conservation of plant genetic resources with a range of new techniques for easy and reliable identification of plant species. Many of these techniques have been successfully used to study the extent and distribution of variation in species gene-pools and to answer typical evolutionary and taxonomic questions [2,3]. Properties desirable for ideal DNA markers include highly polymorphic nature, codominant inheritance (determination of homozygous and heterozygous states of diploid organisms), frequent occurrence in the genome, selective neutral behavior (the DNA sequences of any organism are neutral to environmental conditions or management practices), easy access (availability), easy and fast assay, high reproducibility, and easy exchange of data between laboratories [4].

Sequencing based molecular techniques provide better resolution at intra-genus and above level, while frequency data from markers such as random amplified polymorphic DNA (RAPD), amplified fragment length polymorphism (AFLP) and microsatellites provide the means to classify individuals into nominal genotypic categories and are mostly suitable for intra-species genotypic variation study [5]. This distinction is important to grasp for population studies, particularly when the diversity data are used as a basis for making decisions about conservation of plant resources. For instance, a recent study on Napier grass (*Pennisetum purpureum*) has showed that AFLP is incompatible with RAPD and morphological data; re-registration of all accessions of Napier grass based on DNA barcoding is suggested as a means to resolve the lingering problems regarding the identity of accessions [6]. The main objective of this review is to provide a basic understanding of the recently developed molecular tools and their potential application in the conservation of plant resources.

## 2. DNA Sequencing

DNA sequencing is the determination of the order of the nucleotide bases-A (adenine), G (guanine), C (cytosine) and T (thymine) present in a target molecule of DNA. Early work that was developed for the identification and characterization of clinically important bacterial strains has made it possible to obtain DNA sequences within a few days [7,8]. We describe the conventional and next generation sequencing techniques separately under the following subheadings.

### 2.1. Conventional Sequencing Technique

**Figure 1.**
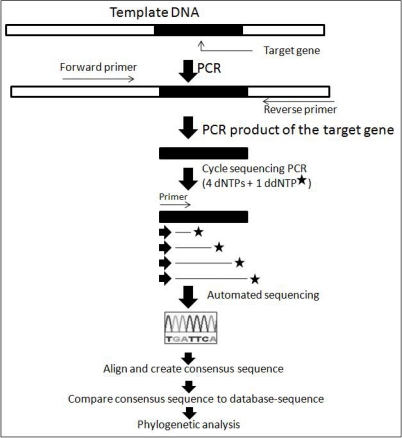
Schematic diagram summarizing the sequencing of a target gene for application in phylogenetic analysis (modified from [10]).

Currently, dye-terminator sequencing technique is the standard method in automated sequencing analysis [9]. The dye-terminator sequencing method, along with automated high-throughput DNA sequence analyzers, is now being used for the vast majority of sequencing work. The basic technique related with dye terminator sequencing and phylogenetic analysis is illustrated in Figure 1. Dye-terminator sequencing utilizes labeling of the chain terminator ddNTPs, which allows sequencing in a single reaction, rather than four reactions as in the previously used labeled-primer method. In dye-terminator sequencing, the four dideoxynucleotide chain terminators are labeled with fluorescent dyes, each with a different wavelength of fluorescence emission. The main advantages of this technique are its robustness, automation and high accuracy (>98%). On the other hand, the limitations of this technique include dye effects due to differences in the incorporation of the dye-labeled chain terminators into the DNA fragment. Such incorporation of dye can result in unequal peak heights and shapes in the electronic DNA sequence trace chromatogram after capillary electrophoresis. Another drawback is its inability to handle long sequences; however, it can reliably sequence up to approximately 900 nucleotide long DNA fragments in a single reaction. The advent of new generation sequencers with solid state chemistry has significantly overcome these problems.

Current interest is in the DNA barcoding of plants with the aim to identify an unknown plant in terms of a known classification. DNA barcoding is a technique for characterizing species of organisms using a short DNA sequence from a standard and agreed-upon position in the genome. DNA barcode sequences are very short relative to the entire genome and they can be obtained reasonably quickly and cheaply [11]. The success of species-level assignment of plants using Basic Local Alignment Search Tool (BLAST) [12] with individual barcodes was obtained with matK (99%), followed by trnH-psbA (95%) and then rbcL (75%). Use of three-locus DNA barcode resulted in >98% correct identifications of 296 species of woody trees, shrubs and palms [13]. Recently, a group of plant DNA barcode researchers proposed two chloroplast genes, rbcL and matK, taken together, as appropriate for barcoding of plants [14].

Molecular phylogenies in plants are traditionally based on chloroplast DNA (cpDNA) sequence variation [15]. This approach has proved to be very powerful at the family level through the sequencing of coding regions such as rbcL [16]. However, low evolutionary rate of these sequences limits the power of cpDNA for the assignment at the genus or species level [17]. As a consequence, the relationships among closely related taxa have been inferred using non-coding sequences [18]. However, the potential problems due to gene flow of cpDNA among closely related taxa, as well as the lack of phylogenetic resolution, triggered the development of new approaches based on nuclear DNA. The most common alternative corresponds to the sequencing of the ITS (internal transcribed spacer) of 18S–25S nuclear ribosomal DNA [19,20]. When both cpDNA and ITS sequencing fail to resolve phylogenies, the amplified fragment length polymorphism (AFLP) approach has the potential to solve such difficulties, particularly among closely related species, or at the intra-specific level [21–23]. Therefore, integration of recently developed barcoding with the following techniques such as RAPD, AFLP, microsatellite and SNP seems to provide better resolution.

### 2.2. Next Generation Sequencing Techniques

**Figure 2.**
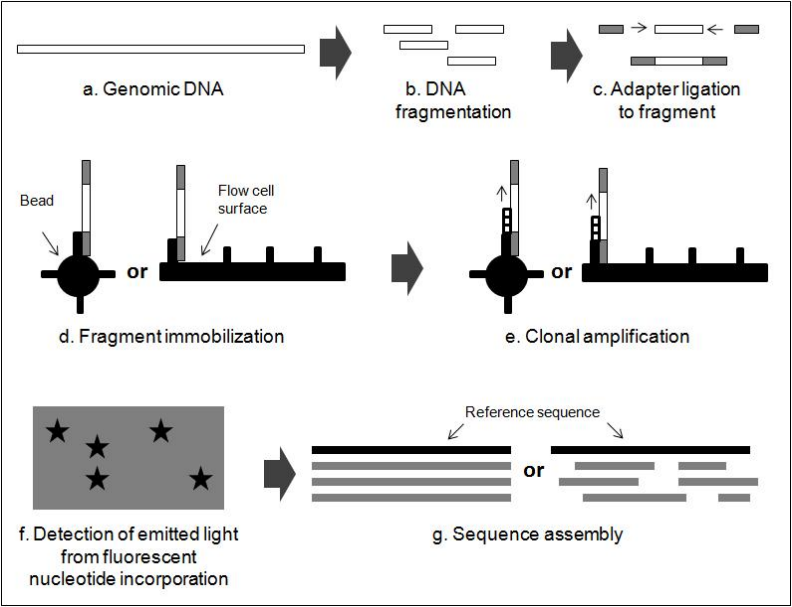
A common workflow of next-generation sequencing methods (modified from [29]).

A new generation of non-Sanger based sequencing technologies has been evolving on its promise of sequencing DNA at unprecedented speed, thereby also having enabled impressive scientific achievements and novel biological applications. These techniques have made it possible to conduct robust population-genetic studies based on complete genomes rather than just short sequences of a single gene. Rapid progress in genome sequences of various plant species through next generation sequencing will further extend our understanding how genotypic variation translates into phenotypic characteristics. A comparative genomic approach is extraordinarily useful for identifying functional loci related to morphological, geographical and physiological variation, and thus next generation sequencing technology will enable us to better understand the process of plant evolution. Next generation platforms do not rely on Sanger chemistry [24] as did the first generation machines used for the last 30 years [25]. The first of this kind of 2nd generation of sequencing technique appeared in 2005 with the landmark publication of the sequencing-by-synthesis technology developed by 454 Life Sciences [26] based on pyrosequencing [27,28]. Commercial 2nd generation sequencing methods can be distinguished by the role of PCR in library preparation. There are four main platforms; all being amplification-based: (i) Roche 454 GS FLX, (ii) Illumina Genome Analyzer IIx, (iii) ABI SOLiD 3 Plus System and (iv) Polonator G.007 [29]. Common principles of these 2nd generation sequencing techniques are illustrated in Figure 2.

The single-molecule sequencing method (also known as 3rd generation or next-next generation) is independent of PCR [25,30]. This mode of sequencing protocol was recently developed by Helicos Genetic Analysis System using the technology developed by Braslavsky *et al*. [31]. Other 3rd generation sequencing systems are being developed by Life Technologies and Pacific Biosciences SMRT technology and may appear within one to two years. Oxford Nanopore Technology (www.nanoporetech.com) has been developing a label-free, electrical, single-molecule genuinely revolutionary DNA sequencing method. This technique is aimed at obviating the need for amplification or labeling by instead detecting a direct electrical signal [32]. However, this technique is still in a developing stage. The recently developed Helicos 3rd generation high-throughput and low-cost direct single molecule RNA sequencing method - without requiring prior conversion of RNA to cDNA - opened the door for a comprehensive and bias-free understanding of transcriptomes [33].

**Figure 3.**
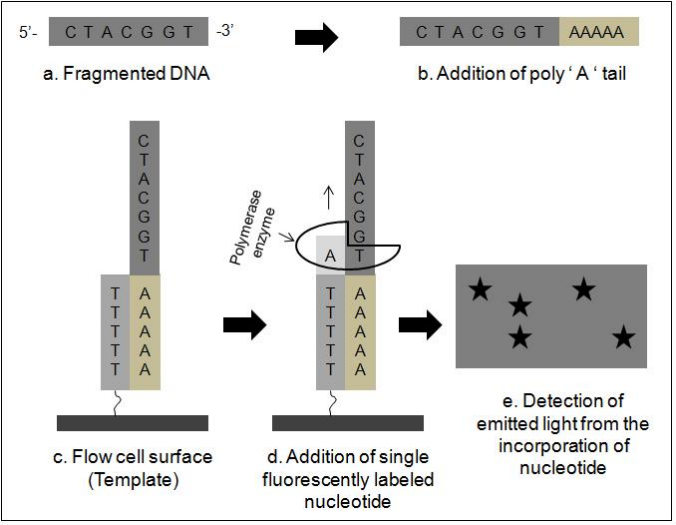
Basic workflow of Helicos single molecule sequencing method (modified from [30]).

By directly sequencing single molecules of DNA or RNA, Helicos True Single Molecule Sequencing (tSMS) technology significantly increased the speed of sequencing, while also decreasing the cost. Briefly, the procedure works by: first capturing billions of single molecules of sample DNA on an application-specific proprietary surface within two flow cells. These captured strands serve as templates for the sequencing-by-synthesis. Polymerase and one fluorescently labeled nucleotide (C/G/A/T) are added. The polymerase catalyzes the sequence-specific incorporation of fluorescent nucleotides into nascent complementary strands on all the templates. After a wash step, which removes all free nucleotides, the incorporated nucleotides are imaged and their positions are recorded. The fluorescent group is removed in a highly efficient cleavage process, leaving behind the incorporated nucleotide. The process continues through each of the other three bases (Figure 3; modified from [30]). Using the Helicos DNA Barcoding protocol, scientists at Helicos were able to multiply the system’s sample throughput five-fold (from 50 samples to 250 samples per run), without compromising accuracy or representational bias [30]. DNA sequencing data from next generation platforms typically present shorter read lengths, higher coverage and different error profiles compared with Sanger sequencing data. Several software packages have been created especially to cope with the next generation sequencing data. A good review on these recent software tools has been published by Miller *et al*. [34].

Since the advent of next generations sequencing, these techniques have been helping to uncover secondary metabolic pathways, to analyze cDNA-array based gene expression, for genetic manipulation to improve yield of desirable secondary products and molecular marker identification. For example, an Expressed Sequence Tag (EST) library from whole plantlets of medicinal plant (*Salvia miltiorrhiza*) was generated with the expression patterns of 14 secondary metabolic enzyme genes in different organs. Additionally, a total of 122 microsatellites were identified from the ESTs, with 89 having sufficient flanking sequences for primer design. This set of ESTs represents a significant proportion of the *S. miltiorrhiza* transcriptome and gives preliminary insights into the gene complement of *S. miltiorrhiza* [35]*,* which was a very laborious task a few years back. Using 454 and Illumina EST sequencing of the parental diploid species of *Tragopogon miscellus* (Moscow salsify, Asteraceae), 7,782 single nucleotide polymorphisms were identified that differ between the two progenitors genomes present in this allotetraploid [36]. Next generation high through-put Solexa sequencing technology led to the discovery of 14 novel and 22 conserved miRNA families from peanut [37]. Recently, a new variety of chickpea (*Cicer arietinum*), resistant to *Helicoverpa armigera* (Pod borer), has been developed with the help of valuable information retrieved from next generation sequencing [38].

## 3. Random Amplified Polymorphic DNA (RAPD)

RAPD is based on the amplification of genomic DNA with single primers of arbitrary nucleotide sequence [39]. These primers detect polymorphisms in the absence of specific nucleotide sequence information and the polymorphisms function as genetic markers and can be used to construct genetic maps. Since most of the RAPD markers are dominant, it is not possible to distinguish whether the amplified DNA segment is heterozygous (two different copies) or homozygous (two identical copies) at a particular locus. In rare cases, co-dominant RAPD markers, observed as different-sized DNA segments amplified from the same locus, may be detected [39].

**Figure 4.**
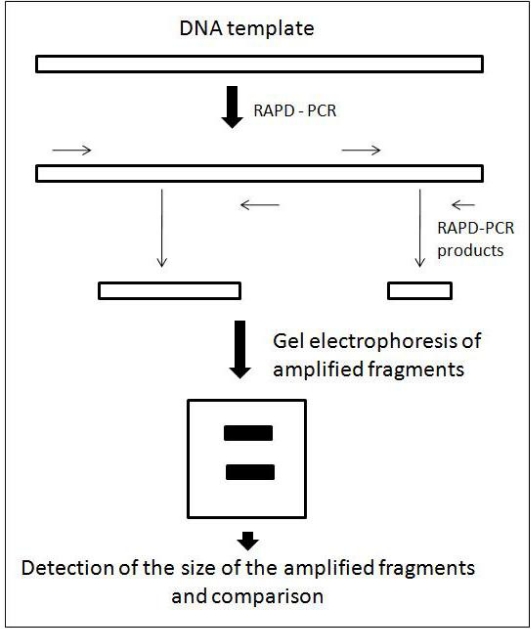
The principle of RAPD-PCR technique. Arrows indicate primer annealing sites (modified from [40]).

The basic technique of RAPD involves (i) extraction of highly pure DNA, (ii) addition of single arbitrary primer, (iii) polymerase chain reaction (PCR), (iv) separation of fragments by gel electrophoresis, (v) visualization of RAPD-PCR fragments after ethidium bromide staining under UV light and (vi) determination of fragment size comparing with known molecular marker with the help of gel analysis software. A diagrammatic presentation of these steps is given in Figure 4. It is important to note that RAPD technique requires maintaining strictly consistent reaction conditions in order to achieve reproducible profiles. In practice, band profiles can be difficult to reproduce between (and even within) laboratories, if personnel, equipment or conditions are changed [3]. Despite these limitations, the enormous attraction of this technique is that there is no requirement for DNA probes or sequence information for primer design. The procedure involves no blotting or hybridizing steps. The technique is quick, simple and efficient and requires only the purchase of a thermocycling machine and agarose gel apparatus and relevant chemicals, which are available as commercial kits (e.g., Ready-To-Go RAPD analysis beads; GE Healthcare, Buckinghamshire, UK). Another advantage is the requirement for only small amounts of DNA (10–100 ng per reaction) [3].

The RAPD markers have been used for detecting genomic variations within and between varieties of sweet potato. A total of 160 primers were tested and eight showed consistent amplified band patterns among the plants with variations within and between varieties [41] of sweet potato. Genetic diversity was evaluated by RAPD markers and morpho-agronomic characters for a total of 42 accessions of Barberton daisy (*Gerbera jamesonii*) employing a set of 12 primer pairs [42]. Germplasm accession of 80 *Plantago* spp. was studied by using RAPD with the help of 20 random primers [43]. Recently, RAPD has been used for estimation of genetic diversity in various endangered plant species [44–47].

## 4. Amplified Fragment Length Polymorphism (AFLP)

**Figure 5.**
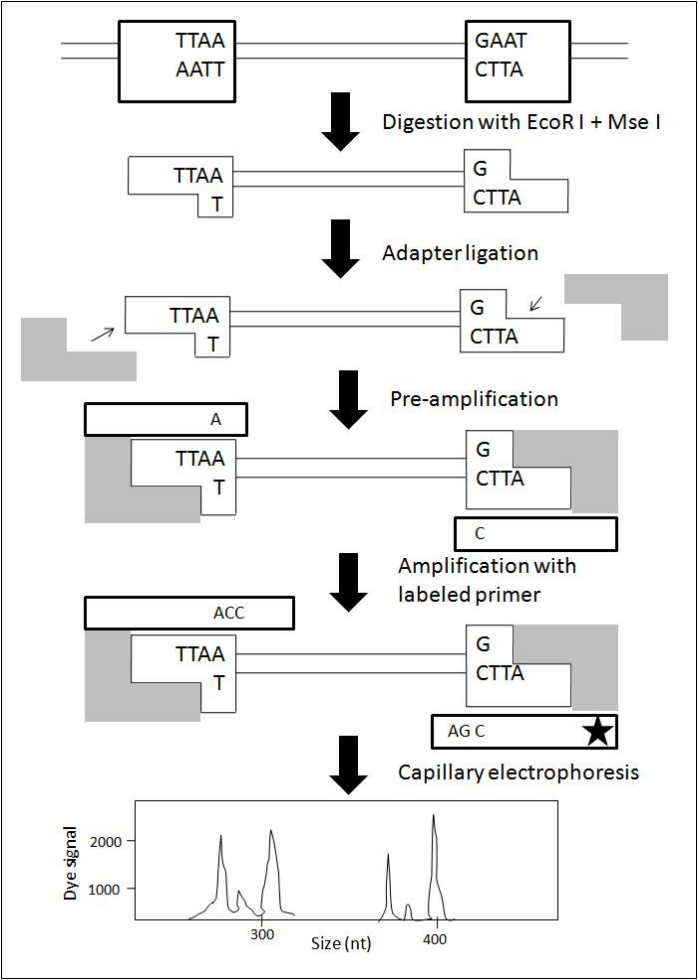
A schematic flow chart showing the principle of the AFLP method (modified from [49]).

The AFLP technique is based on the selective PCR amplification of restriction fragments from a total digest of genomic DNA [48]. The technique involves: (i) extraction of highly purified DNA, (ii) restriction endonuclease digestion of DNA (enzyme mixture, usually EcoRI + MseI), (iii) ligation of adapters (enzyme adapters), (iv) pre-PCR (amplification of the restriction fragments; pre-selective amplification with EcoRI primer + A and MseI primer + C), (v) selective-PCR with labeled primer pair (Primer + 3 base pairs; forward labeled, reverse unlabeled), and (vi) gel electrophoresis and fragment analysis by automated sequencing machine (Figure 5). The electrophoretograms can be analyzed using programs like GeneMapper [50].

AFLP is applicable to all species, and unlike RAPD, this technique is highly reproducible as it combines restriction digestion and PCR. However, AFLP requires more DNA (300–1000 ng per reaction) and is more technically demanding than RAPD, however the automation and recent availability of kits means that this technology can be brought to a higher level [3]. Nuclear and chloroplast sequences sometimes fail to reveal variability when plant species are closely related. However, AFLP distributed throughout the whole genome provides a robust solution to overcome the hurdles in plants fingerprinting [15].

A good review on AFLP markers in surveys of plant diversity is published by Mba and Tohme [51]. Recently, several plants in germplasm collections such as *Jatropha curcas* [52] and *Rhodiola rosea* [53] have been characterized by AFLP. Teyer *et al.* [54] have studied the wild populations of *Agave angustifolia* in the desert using AFLP to measure the genetic variability within and between natural populations. AFLP markers have been extensively used for phylogenetic analysis and determining the genetic diversity for conservation of endangered plant species [55–58].

## 5. Microsatellites

**Figure 6.**
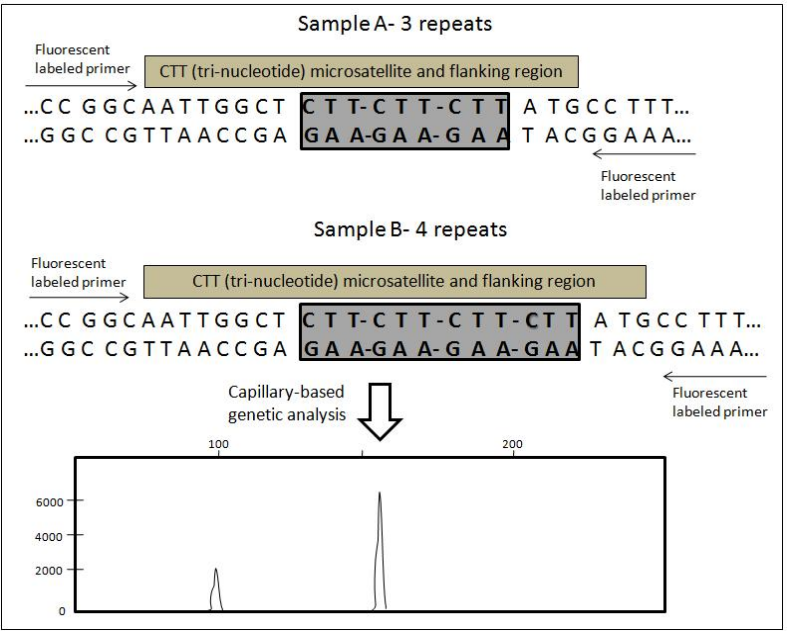
Representation of a CTT (tri-nucleotide) microsatellite and flanking region and the detection method. Arrows indicate positions of PCR primers. Two length variants are shown (A and B) (modified from [61]).

Microsatellites, or simple sequence repeats (SSRs), are polymorphic loci present in DNA that consist of repeating units of one to six base pairs in length [59]. One common example of a microsatellite is a (CA)_n_ repeat, where n is variable among different alleles. These markers often present high levels of inter- and intra-specific polymorphism, particularly when the tandem repeats number is 10 or greater [60]. The repeated sequence is often simple, consisting of two, three or four nucleotides (di-, tri- and tetra- nucleotide repeats) and can be repeated many times. The basic principle of microsatellite is illustrated in Figure 6.

Microsatellites can be amplified for identification by PCR using the unique sequences of flanking regions as primers. The most common way to detect microsatellites is to design PCR primers that are unique to one locus in the genome and that base pair on either side of the repeated portion. Therefore, a single pair of PCR primers will work for every individual in the species and produce different sized products for each of the different length of microsatellites. The PCR products are separated either by slab gel electrophoresis or capillary gel electrophoresis in an automated sequencer.

Microsatellites have proved to be versatile molecular markers, particularly for population analysis, but they are not without limitations. With the abundance of PCR technology, primers that flank microsatellite loci are simple and quick to use, but the development of correctly functioning primers is often a tedious and costly process. Microsatellites developed for particular species can often be applied to closely related species, but the percentage of loci that successfully amplify may decrease with increasing genetic distance [62]. Microsatellite technique has recently been used to establish conservation strategy of endangered plants like *Calystegia soldanella* [63], *Tricyrtis ishiiana* [64] and *Galium catalinense* subspecies *acrispum* [65].

## 6. Single Nucleotide Polymorphism (SNP)

Single nucleotide polymorphism (SNP) is a DNA sequence variation occurring when a single nucleotide (A, T, G or C) differs among members of a species. SNP is the most abundant marker system both in animal and plant genomes and has recently emerged as the new generation molecular markers for various applications. Being binary or co-dominant status, they are able to efficiently discriminate between homozygous and heterozygous alleles. Moreover, unlike microsatellites their power comes not from the number of alleles but from the large number of loci that can be assessed [66]. Once the rare SNPs are discovered in a low diversity species, the genetic population discrimination power can be equivalent to the same number of loci in a genetically diverse species. The more evolutionary conserved nature of SNPs makes them less subject to the problem of homoplasy [67]. Most importantly, SNPs are amenable to high throughput automation, allowing rapid and efficient genotyping of large numbers of samples [68].

**Figure 7.**
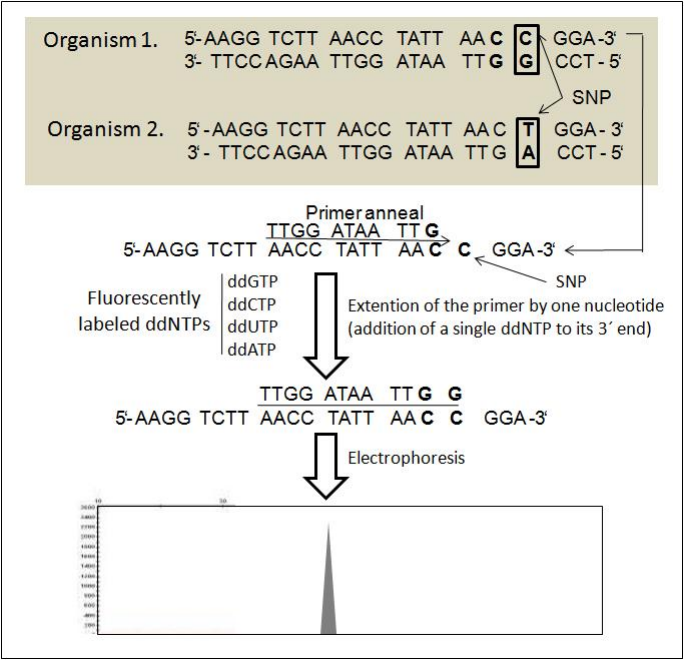
A flow-chart showing the basic principle of SNP method (modified from [75]).

In plants, SNP can be designed from ESTs [69,70] and single-stranded pyrosequencing [71]. A high-throughput genome analysis method called diversity array technology (DArT), based on microarray platform, has been developed for the analysis of plant DNA polymorphism [72]. Eijk *et al*. [73] described a novel SNP genotyping technique, SNPWave. Chip-based SNP arrays use thousands of oligonucleotide probes attached to a solid surface (e.g., glass, silicon wafer) allowing for a large number of SNPs to be interrogated simultaneously [74]. The ABI PRISM SNaPshot Multiplex Kit is designed to interrogate up to 10 single nucleotide polymorphisms (SNPs) at known locations on one to 10 DNA templates in a single tube. The basic principle of SNP and detection method is illustrated in Figure 7. In brief, the protocol includes preparation of sample reactions using template and primer, performing SNaPshot reactions by thermal cycling and conduction of post-extension treatment of the products. Then automated electrophoresis of the samples and finally, analyzing the data.

SNP is able to determine genetic diversity in plants, particularly in species with limited genetic diversity. Determination of population genetic structure of Castor bean (*Ricinus communis*) using SNPs from genome-wide comparisons showed low levels of genetic diversity and mixing of genotypes, leading to minimal geographic structuring of castor bean populations worldwide [66]. Clustering of Castor bean indicated five main groups worldwide and a repeated pattern of mixed genotypes in most countries; most molecular variance occurred within-populations (74%) followed by 22% among-populations and 4% among-continents [66]. Recently, a single nucleotide primer extension (SNuPE) assay targeting gyrB gene has been developed to identify bacteria belonging to the *Burkholderia cepacia* complex, which are very difficult to identify using commonly used phenotypic and molecular techniques [76]. Novel SNP based technique allowed the successful detection and distinction of specific genetic variations and is effectively applied in routine medical diagnosis since it permits to analyze routinely many samples in a short time [77]. Similar approaches need to be utilized in plants that are difficult to discriminate for low level of genetic diversity.

## 7. Concluding Remarks

Molecular markers are indispensable tools for measuring the diversity of plant species. Low assay cost, affordable hardware, throughput, convenience and ease of assay development and automation are important factors when choosing a technology [78,79]. Databases based on a large number of potential characters are readily available for inferring relationships using sequence data. Further advantage of sequencing includes substitutions within structural genes that produce differentiation from changes in morphology [80]. Information from the sequences themselves can be useful for specifying parameters of the model of sequence evolution, which in turn, influences the topology of the inferred tree. To date, next generation sequencing technologies have been applied in a variety of contexts, including whole-genome sequencing, targeted resequencing, discovery of transcription factor binding sites and noncoding RNA expression profiling [81]. A disadvantage of sequencing includes inferences of positional homology that are frequently more problematic for non-coding nucleotide sequences because penalties for insertion-deletion events determine the extent of sequence similarity during pairwise and multiple-alignment. Another potential shortcoming of sequencing is that the evolutionary history of species can be inconsistent with the genealogy of a single gene [82] and it is not possible to assess if a tree topology based on a single gene sequence is likely to represent the original genealogy of the species [83]. The advantages of RAPD include its simplicity, low cost, rapid, use of arbitrary primers, no need of initial genetic or genomic information, and the requirement of only tiny quantities of target DNA. Disadvantages of this technique are dominant type and the lack of a prior knowledge on the identity of the amplification products which in turn creates problems with reproducibility and co-migration [84,85]. The major advantage of the AFLP technique is the large number of polymorphisms that the method generates compared with other markers. The ability of AFLP to differentiate individuals in a population makes the technique useful for paternity analyses [86], gene-flow experiments and also for plant variety registration [87]. However, the methodology of AFLP experiment and post-run data analysis are complex and time consuming compared with other markers like RAPD. The great advantage of microsatellite analysis is the large number of polymorphisms that the method reveals. The ability of the method to differentiate individuals when a combination of loci is examined makes the technique very useful for gene-flow experiments, cultivar identification and paternity analyses [88]. Major problem with the microsatellite relates with the initial screening of an organism for microsatellite library creation [5]. The distribution and frequencies of SNPs are the key factors to understand molecular diversity between closely related populations and species [89]. SNP is potentially useful for the analysis of degraded samples by use of short amplicons and low mutation rates [90]. However, this technique has bi-allelic nature and less resolution power compared with multi-allelic microsatellites [91]; though this shortcoming is overcome by its inherent capacity of scanning a large number of loci. SNP markers are best for characterizing and conserving the gene bank materials and the AFLP and microsatellite markers are more suitable for diversity analysis and fingerprinting [92].

Unfortunately, an ideal marker does not exist for use in all studies; rather a technique or techniques will be suited to a range of investigations. In this context, we agree with Robinson and Harris [5] and conclude that RAPD, AFLP and microsatellites should not be considered appropriate for phylogenetic analyses above the species level. These markers are undoubtedly valuable tools for addressing population genetics and plant breeding issues, but for phylogeny reconstruction and taxonomy they could be problematic and sometimes even misleading, so they must be used with caution. Molecular genetics is a fast-moving field and new techniques are likely to be developed in the near future which will have their own strengths and limitations. Development of molecular technique based on error free database is another essential demand for easy assignment of unknown plant samples into appropriate taxa.
